# Scaffold Characteristics for Functional Hollow Organ Regeneration

**DOI:** 10.3390/ma3010241

**Published:** 2010-01-08

**Authors:** Maya Horst, Srinivas Madduri, Rita Gobet, Tullio Sulser, Heike Hall, Daniel Eberli

**Affiliations:** 1 Laboratory for Urologic Tissue Engineering and Stem Cell Therapy, Department of Urology, University Hospital, Zurich, Switzerland; E-Mails: maya.horst@usz.ch (M.H.); sirinivas.madduri@usz.ch (S.M.); tullio.sulser@usz.ch (T.S.); 2 Cells and Biomaterials, Department of Materials, ETH Zurich, Switzerland; E-Mail: heike.hall@mat.ethz.ch (H.H.); 3 Division of Pediatric Urology, Department of Pediatric Surgery, University Children‘s Hospital, Zurich, Switzerland; E-Mail: rita.gobet@kispi.uzh.ch (R.G.)

**Keywords:** scaffold, regeneration, tissue engineering, biomaterials, hollow organs, regenerative medicine

## Abstract

Many medical conditions require surgical reconstruction of hollow organs. Tissue engineering of organs and tissues is a promising new technique without harvest site morbidity. An ideal biomaterial should be biocompatible, support tissue formation and provide adequate structural support. It should degrade gradually and provide an environment allowing for cell-cell interaction, adhesion, proliferation, migration, and differentiation. Although tissue formation is feasible, functionality has never been demonstrated. Mainly the lack of proper innervation and vascularisation are hindering contractility and normal function. In this chapter we critically review the current state of engineering hollow organs with a special focus on innervation and vascularisation.

## 1. Introduction

The replacement of small intestine and bladder with functional tissue engineered constructs would represent a substantial progress in surgical treatment. In the small bowel, the loss of more than two thirds of the small intestine causes short bowel syndrome (SBS) resulting in life threatening nutritional malabsorption. The most common cause of SBS is extensive surgical resection of the small intestine due to necrotizing enterocolitis in neonates and Cohen’s disease in adults. Therapeutic options available for patients with SBS are aimed at ensuring an adequate supply of nutrients, water, electrolytes, trace elements and vitamins. Currently the therapy includes parenteral nutrition, surgical intestinal lengthening procedures or small bowel transplantation, but all these approaches have limitations.

Constructs for bladder replacement are needed in many medical conditions including severe bladder dysfunction, trauma and cancer. Bladder dysfunction can be induced by disease or surgical intervention altering the normal pattern of storage and voiding. This can result in chronic urinary incontinence and increased upper urinary tract pressure leading to irreversible kidney damage. If conservative treatment fails the treatment of choice in these patients is surgical enlargement of the bladder using intestinal tissue with the aim to increase bladder capacity and lower the storage pressure. Unfortunately, this procedure fails to restore the emptying function and is associated with numerous complications such as metabolic disturbance, increased mucus production, urolithiasis, infections and even malignant diseases. In both, SBS and bladder failure, the current treatments are accompanied by severe morbidity and mortality.

Tissue engineering (TE), one of the major approaches of regenerative medicine, is a rapidly growing and exciting field of research. In combination with better understanding of structure, biology and physiology cell culture techniques or TE may offer new treatment options for patients needing replacement or repair of an organ. The principle is to dissociate cells from a tissue biopsy, to expand these cells in culture, and to seed them onto the scaffold material *in vitro* in order to form a live tissue construct prior to reimplantation into the recipient’s organism. In the appropriate biochemical and biomechanical environment these tissues will achieve their full functional potential and serve as native tissue equivalents. The TE approach has major advantages over traditional organ transplantation. Tissues that closely match the patient’s needs can be reconstructed from a generally readily obtainable biopsy. Moreover the new reconstruct can be transplanted into the patient’s body without donor site morbidity and with minimal or no immunogenicity. This eventually conquers several limitations, encountered in tissue transplantation approaches.

The concept of tissue engineering has been applied clinically for a variety of disorders, for example artificial skin for burn patients [1], tissue engineered trachea [2], cartilage for knee-replacement procedures [3], injectable chondrocytes for the treatment of vesico-ureteric reflux [4] and urinary incontinence [5,6].

For hollow organs such as bladder, intestine, as well as urethra, oesophagus, vagina, or blood vessels the strategies generally include implantation of either a biomaterial, which subsequently becomes integrated into the host organism through immigration of cells from surrounding tissues, or, with the technical advances in TE preferably an *in vitro* precombined construct of materials and cells. The basic requirements to achieve functional tissue are appropriate *cells*, a suitable *environment* and proper *scaffolds*.

Recently, several studies reported the use of tissue-engineered small intestine in small animal models using natural or synthetic polymers scaffolds seeded with autologous cells [7,8]. Reports on large animal models (dog and pig) were able to demonstrate the feasibility of successful generation of engineered intestine with correct architecture, including features of an intact stem cell niche [9,10]. Even if the histology of engineered intestinal segments show normal structures including the presence of neuronal elements and appear to be functional, motility has not yet been demonstrated in these segments.

For bladder TE, several studies have confirmed feasibility of bladder reconstruction using engineered segments which were formed using biomaterials seeded with autologous cells *in vitro* [11,12]. This basic approach was first examined in a canine subtotal cystectomy model, where bladder constructs configured from natural or synthetic scaffolds and seeded with expanded autologous bladder-derived cells were successfully implanted [11]. This approach provided improved findings when compared with earlier attempts. Encouraged by the promising results of several animal studies, a similar approach was applied more recently to a small series of patients with severe neuropathic bladder dysfunction by Atala *et al*. in 2006 [13]. They reported the results of seven patients aged 4 to 19 years, with a mean follow up of four years. All three patients with the engineered tissues made with composite-scaffolds (collagen-PGA) showed a significant increase in bladder capacity and compliance. Tissue biopsies of the engineered bladder segments were described as showing an adequate structural architecture and phenotype of the different cells. Although some patients benefit was reported, clinical improvement was minimal. So far efficacy of this novel approach does not compare favorable with conventional reconstruction methods using bowel segments as source of material. To date, the clinical application of engineered tissues has been hampered by slow vascularization, poor nutrition leading to cell death and consecutive tissue fibrosis [12,14]. For successful bladder reconstruction revascularization of the construct is essential to support short and long term survival. Furthermore, to restore physiological bladder function reinnervation is indispensable.

The ambitious goal to reconstruct a functional, contractile bladder responsive to voluntary control is an ongoing challenge for the future. Our ability to use donor tissue efficiently, to provide the optimal conditions for cultivation, long-term survival, differentiation and growth will lay the foundation for success.

## 2. Cells

The basic bricks of living organisms, the cells, are a predominant factor for successful TE. Tissue renewal requires an adequate number of regeneration-competent cells that do not elicit immune response. Therefore autologous cells are the ideal choice, as their use circumvents many of the inflammatory and rejection issues associated with a nonself donor approach [28]. With the past decade, major advances in the expansion of a variety of primary human cells have been achieved. The different types of cells commonly used for TE applications can be categorized as differentiated non-stem or adult cells and as undifferentiated stem cells. Each of these cell types brings along its own advantages and disadvantages.

The epithelium of the intestine is a confluent cell layer consisting of a number of cell subtypes such as enterocytes, Goblet cells, enteroendocrine cells as well as intestinal stem cells [29]. It has been shown in intestinal organoids (multicellular units derived from intestine) that disaggregated intestinal tissue containing all the different cell types and mesenchymal stroma, facilitate the generation of neo-intestine [30,31]. Furthermore, intestinal organoids have been shown to be superior to isolated intestinal cells for seeding the biomaterial [29].

In the field of bladder reconstruction adult urothelial and smooth muscle cells can be efficiently harvested from biopsy material, expanded extensively in culture and reimplanted into the same host [12,32,33]. Their differentiation characteristics, growth requirements and other biological properties have been widely studied [34,35,36]. However, for elderly patients or patients with extensive end stage organ failure, a tissue biopsy may not yield enough normal cells for expansion and transplantation. Therefore adult progenitor cells may not be sufficient or appropriate for tissue engineering and transplantation in all cases [37].

Although adult progenitors remain important for TE, the use of pluripotent stem cells has recently been recognized as a promising alternative source of cells from which the desired tissue can be derived. Stem cells, by definition, are immature or undifferentiated cells with the potential to go through numerous cycles of self-renewal, thus replenishing the pool of stem cells as well as to differentiate into more specialized, tissue-/organ-specific cells. Stem cells can be categorized into embryonic, fetal or adult cells according to their source of origination (NIH).

**Embryonic stem cells** exhibit two remarkable properties: the ability to proliferate in an undifferentiated, but still pluripotent state (self-renewal), and the ability to differentiate into a large number of specified cells [38]. As their name implies, embryonic stem cells are derived from the early stage embryo. Although embryonic stem cells research is thought to have much greater potential than adult stem cells, several ethical and legal controversies still exist concerning their use in humans. Furthermore, embryonic stem cells have been shown to transdifferentiate into a malignant phenotype, forming teratomas [39,40].

**Fetal stem cells** derived form amniotic fluid and placentas have recently been described and represent a novel source of stem cells [41]. The principle stem cell type isolated from amniotic fluid and placentas is mesenchymal, but they have an expansion potential that is superior to that of adult stem cells. They are less immunogenic as they do not express human leukocyte antigen (HLA) and they do not form teratomas *in vivo*. Fetal stem cells are multipotent and they have been shown do differentiate into myogenic, adipogenic, osteogenic, nephrogenic, neural, and endothelial cells. In addition, the cells have a high replicative potential and could be stored for future use, without the risk of rejection and without ethical concerns [42].

**Adult stem cells** are basically undifferentiated cells found among differentiated cells in a tissue or organs. They are present in all adult tissues and are critical to tissue health, maintenance, and response to injury or disease throughout life. When compared with embryonic stem cells adult stem cells are more committed but still have the plasticity to differentiate into all three germ layers [43]. However, they demonstrate considerable advantages, including; stable differentiation into specific cell lineages, no transdifferentiation into a malignant phenotype (teratomas), no requirement for the sacrifice of human embryos for their isolation and no or little immune rejection. Furthermore, certain ethical and legal issues can also be overcome.

Today, pluripotent stem cells, or differentiable adult stem cells, can be harvested from many different tissues, including bone marrow [44,45] striated muscle [46] fat [47], skin [48], synovial membrane [49], and, more recently, testicles [50,51]. These cells can differentiate into committed cells of other tissues, a feature defined as plasticity. This would allow for engineering of composite tissues composed of multiple cell types using one single source of adult stem cells. Therefore, adult stem cells are particularly suitable for cellular therapy and for the engineering of tissues and organs.

Bladder reconstruction using stem cells seeded on a scaffold has recently been shown to be a promising alternative for bladder engineering [41,52,53,54,55]. Recent progress suggests that engineered tissues and cell based therapies using adult stem cells may have an expanded clinical applicability in the future and may represent a viable therapeutic option for those who require tissue replacement or repair.

The most intensively investigated adult stem cells are mesenchymal stem cells (MSCs). This cell type holds significant promise for the engineering of musculoskeletal structures. Bone marrow represents the major source of MSCs. Chung *et al*. performed studies in a rat model that examined the ability of MSCs to aid in the regeneration of bladder tissue on an acellular matrix scaffold [52]. The cells were seeded onto an acellular matrix (small intestinal submucosa) and used in bladder augmentation studies. The number of smooth muscle containing bundles was dramatically increased in the seeded grafts versus the unseeded controls, suggesting that the mesenchymal stem cells (MSCs) have differentiated into smooth muscle cells (SMCs) and have contributed to the regeneration of the graft. In a similar study Zhang *et al*. described the isolation and expansion of bone marrow MSCs from dogs for use as an alternative cellular source for autologous bladder grafts using small intestinal submucosa (SIS) [53]. These authors demonstrated the ability of MSCs to differentiate into SMCs and provide a contractile force on collagen matrices *in vitro*. MSCs also enhanced the regenerative process when seeded onto SIS for augmentation cystoplasty by enhancing smooth muscle bundle formation [53]. However, in both studies, the cells were not labeled and therefore were indistinguishable from cells that may have migrated in from the surrounding normal tissue.

Another source of adult stem cells is fat tissue. Unlike bone marrow stem cells, which are difficult to isolate and relatively scarce, adipose stem cells (ASCs) are tremendously abundant and easily accessible [56]. Jack *et al*. demonstrated the feasibility of bladder tissue engineered from adipose stem cells. They showed that ASCs differentiated into bladder smooth muscle cells and showed contractile function *in vivo*. The vast availability of ASCs combined with their ease of procurement and ability to differentiate into contractile smooth muscle make them a competitive non-embryonic alternative for regeneration of the bladder and other smooth muscle tissues [56].

Although stem cells are believed to be the key factor for the future of TE, one of the major challenges associated with the use of these cells is to provide appropriate cellular environment cues that regulate cell growth and subsequent tissue formation in a controlled and efficient manner [27]. A deeper understanding of the complex interplay of stem cells and environment might allow for new strategies where stem cells actively participate in functional tissue and organ formation. Stem cells will differentiate into various cell types in situ depending on the requirements of the regenerating tissue, or they remain stem cells that generate progeny to maintain the tissue. In addition stem cells could be used to secrete factors that enhance cellular ingrowth, neo-vascularisation, and re-innervation. Intestinal organoids that grew on scaffolds not only repopulated the intestine but also resulted in vascular and lymphatic proliferation, predominantly through vascular endothelial growth factor (VEGF) production [57].

## 3. Environment

Successful TE approaches depend on meeting a variety of critical experimental conditions. One is to create an environment conductive to cell growth, differentiation and eventually enabling the integration of an implanted TE-construct with the surrounding host tissue. In order to achieve this, the TE-constructs shouldn’t induce an immune response *i.e.,* the host cells do not recognize it as a foreign body. Furthermore TE-constructs aim to mimique mechanical and biochemical properties of the native extracellular matrix (ECM). The ECM is the optimized natural milieu that directs tissue development and maintains tissue homeostasis. The ECM refers to a complex network of molecules that provide 2D- or 3D-mechanical support for cells serves as a barrier between different compartments or cell types and provides guidance cues during development, tissue repair or wound healing. On the individual cell basis ECM induces cell polarity, allows or inhibits cell adhesion, promotes or slows down migration and induces cell and tissue differentiation. It might also induce programmed cell death [18]. The ECM is composed of chemically very different macromolecules that are assembled into organized structures remaining in close association with the surface of the cells that secreted them. The main components are space filling proteoglycans, containing collagen fibers and non-collagenous glycoproteins such as elastin. Integrated into this hydrogel-like matrix are signaling molecules such as growth factors, cytokines and hormones [19,20]. The ECM occurs in many different forms depending on the requirements of the surrounding tissue. In many cases it is a 3D-strucuture of ECM surrounding cells which maintains the tissue specific 3D-architecture. In other cases ECM forms flexible sheet-like structures between 40–120 nm thickness that serve as solid support layers composed of network forming laminin-entactin complexes, type IV collagen and heapran sulphate proteoglycans. This sheet-like ECM called basal lamina is frequently found in hollow organs such as blood vessels or bladder tissue. The ECM is tissue specific and the components self assemble to form spontaneous 2D- or 3D-structures under physiological conditions. Therefore, a great effort has been made to understand the appropriate biological, physical, and chemical cues, with the aim to mimic the ECM to guide morphogenesis in tissue repair [21].

Moreover, the ECM is constantly changing in composition and structure as tissues develop, remodel, repair, and age [22]. All body cells, except the blood cells interact directly and in a very specific manner with their surrounding ECM. Specific receptor-ligand contacts are established that enable mutual communication between the ECM and the interior of the cell thus regulating matrix assembly, specific remodeling and local removal or disassembly of the matrix [21]. Cell-matrix contacts are mainly formed between different integrins assembled into giant transmembrane protein complexes that regulate and specify their ligand binding affinity as well as matrix assembly. Integrins are transmembrane heterodimeric glycoproteins consisting of one α and one β subunit forming at least 24 integrin-heterodimers known in humans. Many integrins require divalent cations (Ca^2+^, Mg^2+^ or Mn^2+^) for structural integrity and ligand binding as well as activation through cluster formation in order to be fully functional [23,24,25,26]. Although many cell-matrix contacts are formed and enable the cell to respond to their immediate 2D- or 3D-environment, these contacts are transient and strongly regulated. After implantation of a TE-construct cell-matrix interactions are governed by surface properties of the scaffold material and their imperative interactions found to occur. In addition to these cell-matrix interactions several growth factors and biological molecules are also involved in cell adhesion, cell-cell communication and cell-matrix interaction. This very complex field was covered by recent reviews ([27]), and will therefore not be further discussed in this chapter.

## 4. Biomaterials

The purpose of the scaffold is to serve as a temporary supporting structure allowing not only 3-dimensional support of tissue growth and formation but also providing the biological environment needed for cellular growth, differentiation and tissue formation. Scaffold materials can be of natural or synthetic origin. The underlying principle for the design of scaffolds for TE is similar for different types of tissues. The scaffold preferably mimics the structure and biological functions of native ECM, both in terms of chemical composition and physical properties. Native ECM is a complex and dynamic environment filled with nano-features such as fibers displaying a certain pore size and interconnectivity that should ideally exhibit tissue-specific structures and properties. When naturally derived scaffolds are used they provide specific ligands for cell adhesion and migration, as well as various growth factors inducing specific cell proliferation and functions.

In the early days stiff or non-compliant materials have been investigated for their use in tissue engineering. These materials were not suitable to support the formation of healthy tissue due to biomechanical failure and biological incompatibility [15,16,17].

Currently, a variety of materials are available for manufacturing scaffolds for TE, including native and synthetic polymers and their composites. The choice of material depends on the type of tissue to be reconstructed. Most hollow organs are organized in a similar fashion, consisting of epithelium surrounded by a collagen type I-rich connective tissue and a smooth muscle layer. The epithelial or endothelial layer serves as a barrier preventing the content of the lumen from permeating into the body. The collagen type-I rich layer and the muscle layer maintain the structural and functional integrity of the organ. The cells within these layers interact with each other and with structural proteins to regulate cellular differentiation and function [58].

The following characteristics are desirable for scaffolds used for TE in general. The scaffold should:

- be biocompatible, meaning that it should not provoke any rejection, inflammation, immune responses or foreign body reactions.
- provide a 3D template for the cells to attach and to guide their growth.
- have a porous architecture with a high surface area for the maximum loading of cells, cell-surface interaction, tissue ingrowth, and transportation of nutrients and oxygen.
- be degradable under physiological conditions and the degradation rate should match the rate of tissue regeneration to sustain tissue functionality.
- be mechanically strong to withstand *in vivo* biological forces.
- support the cells in synthesizing tissue specific extracellular matrix components and growth factors required for healthy tissue growth.
- be sterilizable to avoid toxic contaminations without compromising any structural and mechanical properties.

Finally, the production process of the scaffold with all the above unique characteristics must be accomplished in a reproducible, economical, and up-scalable manner [27].

In respect to hollow organs the ideal scaffold material should

- provide structural support for distinct cell layers, including an adequate surface for stable attachment of urothelial cells.
- give adequate biomechanical support to harbor a high density of smooth muscle cells on the exterior surface without inducing premature collapse of the hollow organ.
- serve as a barrier between luminal contents and the body cavity.
- support the formation of unidirectional muscle tissue in defined layers and allow for rapid innervation and vascularisation.

Since each of the different cell types favors different conditions for optimal growth and differentiation, tissue engineering using multiple cell types must take these factors into account.

Two main classes of biomaterials have been utilized for the engineering of hollow organs; acellular matrices derived from donor tissues, (e.g., bladder submucosa and small intestinal submucosa), and synthetic polymers such as polyglycolic acid (PGA), polylactic acid (PLA), and poly(lactic-co-glycolic acid) (PLGA) (see Table 1). These materials have been tested in respect to their biocompatibility in the host tissues [59,60]. Both types of material have been shown to be able to support the formation of urogenital and gastrointestinal tissue. Acellular tissue matrices are extracted from native tissue and therefore contain growth factors, hormones and other signaling factors [61,62] that promote tissue development and have adhesion domain sequences (e.g., RGD) that may support the phenotype and activity of many types of cells [63]. These matrices are known to slowly degrade upon implantation and are usually replaced and remodeled by ECM proteins synthesized and secreted by transplanted or ingrowing cells [64,65,66,67]. In contrast, synthetic polymers can be manufactured reproducibly on a large scale with controlled properties of their strength, degradation rate and ultra structure [68,69]. Both classes of biomaterials have been used either with or without cells for the tissue engineering of hollow organs, including bladder [11,12,70], urethra [71,72], oesophagus [73], intestine [8], vagina [74], or blood vessels [ 75,76].

**Table 1:** Comparison between the two main classes of biomaterials utilized for TE of hollow organs.

Natually derived scaffolds	Synthetic scaffolds
eg. Bladder submucosa	eg. PGA
+ Mainly collagen, naturally derived	+ Absorbable
+ Absorbable	+ High porosity (up to 95%)
+ Cell recognition sites	+ Low variability
+ Growth Factors	- Synthetic, no recognition sites
- Low elasticity	
- High variability	

### 4.1. Native Acellular Matrices

Native acellular matrices are pioneering materials and offer many potential advantages over synthetic scaffold materials [77]. These collagen-rich matrices are extracted from native tissue by mechanical or chemical decellularization [78,79]. They are either derived from bladder [bladder acellular matrix (BAM)] [80] or from small intestine (SIS) [72]. The tensile backbone of the scaffold consists of fibrillar collagen type I, and the basement membrane, serving as a cyto- and tissue-compatible polymeric scaffold for recellularisation. One important advantage over synthetic materials is the composition of the acellular matrix, including structural and functional proteins found in the native extracellular matrix. Acellular matrix therefore may retain their biological activity. They provide specific integrin binding sites and contain endogenous growth factors encouraging the in-growth of tissue [81]. Furthermore, given that the composition and structure of the ECM is unique to individual tissues, there may be advantages in orthotopic-derived matrices: BAM may be expected to contain more appropriate growth factors for bladder TE than SIS [82]. Once implanted into the body, they slowly degrade supporting the ingrowth of host cells which then start to produce new ECM proteins.

For reconstruction of small intestine SIS has been used unseeded and seeded with intestinal organoids [31], mesenchymal stem cells [83] and smooth muscle cells [9]. In the use of unseeded SIS grafts significant contraction of the implant (20–40%) was observed [8,84]. Ansaloni *et al.* showed mucosal epithelial regeneration, in 2 layers organized smooth muscle fibers, neovascularisation and reinnervation of the regenerated bowel [8]. Research using SIS and cells indicate that the use of appropriate progenitor cells is of great advantage to initiate intestinal tissue regeneration and to reduce tissue shrinkage [9,31,83].

In bladder reconstruction acellular matrices have been used either as a graft alone or seeded with urothelial and smooth muscle cells [11,12,85,86,87]. Analysis of unseeded SIS patches after implantation in dogs showed replacement by normal bladder tissue, vascularisation and re-innervation [70]. However, successful bladder regeneration using SIS appears to be dependent on the revascularization rate of the graft and the extent of the original bladder damage. SIS was not able to support functional tissue regeneration when used in animals with inflamed and contracted bladder remnants [87].

Implanting BAM into the bladder of rats, rabbits, dogs and pigs resulted in the regeneration of urothelial and muscle layers with innervation and vascularisation of the graft [85,86]. Moreover, BAM was shown to release exogenous basic fibroblast growth factor (bFGF) in a rat model of bladder augmentation. bFGF is an important growth factor supporting tissue formation and reducing graft shrinkage [88].

However, problems with poor vascularisation, graft shrinkage and incomplete or disorganized smooth muscle development have been associated with the use of decellularised matrices in the different tissues [87,89,90]. Graft shrinkage occurs due to the fast ingrowth of fibroblasts [89]. It seems to be a frequent finding of a scaffold when used as a graft for hollow structures. Researchers agree that the larger the graft, the more pronounced the shrinkage due to the activity of smooth muscle actin-positive fibroblasts [89,90]. Omental coverage, endothelial cell seeding, or application of exogenous angiogenic growth factors were reported to allow ingrowth of capillaries to the graft [88,91]. However, establishment and maintenance of a permanent and sufficiently robust vascular supply to sustain a large graft for the human bladder remains to be demonstrated.

The above mentioned problems led to the concept of *ex vivo* seeding of autologous cells onto different scaffold materials, with the aim of enhancing tissue integration following implantation. This would minimize the inflammatory response toward the matrix, thus avoiding graft contracture and shrinkage. Yoo *et al*. showed that there was a major difference between BAM used with autologous cells and matrices used without cells [11].

A major disadvantage of these systems is the routine variability in protein composition among the batches. There may also be ethical issues regarding their availability, although most naturally derived scaffolds are porcine xenografts.

### 4.2. Synthetic Polymers

Historically the attempt to incorporate of synthetic materials alone in hollow organs has mostly failed, primarily as a result of biological and mechanical incompatibilities. The initial reports on the use of prosthetic materials, such as Dacron [92] or polytetrafluoroehtylene (PTFE) [93] to repair intestinal defects were unsuccessful. These materials were not able to sufficiently support the neo-tissue formation. The use of absorbable biomaterials such as polyglycolic acid PGA and PLA, seeded with intestinal organoids showed the ability to grow neointestine that has the histological appearance of small bowel and has been anastomosed to the native bowel without causing problems [94].

Amongst others polyvinyl sponges, silicone, polytetrafluoroethylene (Teflon) and resin-sprayed paper have been used to reconstruct the bladder with variable results, but none of the methods have been pursued to the present day [15,16,17]. Modern synthetic polymers such as PGA, PLA and PLGA are widely used in tissue engineering and have been applied in bladder reconstruction. These polymers received FDA approval for a variety of applications in humans, including suture material. The ester bonds in these polymers are hydrolytically labile, thus allowing degradation by non enzymatic hydrolysis.

The degradation of PGA, PLA, and PLGA is accompanied by the release of acidic by-products that lower the local pH and induce a mild foreign body reaction. These natural metabolites are eventually eliminated from the body in the form of carbon dioxide and water over time [95]. The degradation rate of these polymers can be tailored from several weeks to several years by altering the crystalinity, initial molecular weight, and the copolymer ratio of lactic to glycolic acids. Since these polymers are thermoplastics, they can be easily formed into a 3D scaffold with a desired microstructure, gross shape, and dimension by various techniques, including molding, extrusion [96], solvent casting [97], phase separation techniques, gas foaming techniques [98] and electrospinning [99,100]. Many applications in tissue engineering require a scaffold with high porosity and high ratio of surface area to volume. Other biodegradable synthetic polymers, including poly(anhydrides) and poly(ortho-esters) can also be used to fabricate scaffolds for tissue engineering with controlled properties [101].

Compared to natural materials, the advantage of producing a synthetic scaffold material is the full control over processing properties such as strength, biodegradability, microstructure and permeability, however, a fundamental feature of these materials is that they lack the natural signals that regulate cell attachment, growth and differentiation [102,103].

Atala and colleagues first demonstrated the feasibility of cells seeding onto a purely synthetic matrix for implantation *in vivo* [12]. PLGA is a well characterized biomaterial with predictable degradation properties, which is widely used as Vicryl® sutures and meshes. It is non-toxic and biocompatible with both urothelial and bladder smooth muscle cells [59,60]. These qualities make PLGA an attractive candidate for combination with natural materials to form implantable constructs for bladder reconstruction. Oberpenning *et al.* also used PGA meshes, molded into the shape of a bladder and surface-coated with PLGA. The constructs were seeded with autologous smooth muscle cells on the outer and urothelial cells on the inner surfaces of the scaffold material [12]. After subtotal cystectomy in dogs the bladder constructs were then implanted onto the bladder base (trigone) and the neo-bladder was then coated with fibrin glue and surrounded with omentum. The animals were monitored for up to 11 months. There were no complications and at three months, the polymer had degraded. Functionally, the reconstructed bladders provided an adequate capacity with good compliance. Histologically and immunocytochemically, the bladders showed an adequate structural architecture, and phenotypically, the urothelium and muscle retained their program of normal differentiation.

As previously mentioned, synthetic biodegradable polymers lack the presence of extra-cellular matrix components, and therefore of cell adhesion sequences and signaling molecules. However, modification of the polymer scaffolds’ chemistry and the method of manufacturing allow improvement of cell adherence potential, growth rate and phenotype regulation [104,105]. It is essential that the structural features of the produced scaffolds resemble the natural ECM in order to provide tissue formation and promote rapid clinical translation. Recently, numerous investigations have explored the possibility of producing scaffolds similar to natural ECM [106,107]. These scaffolds possess a high surface area, high porosity, small pore size, and a low density, all of which are features essential for the improvement of cell adhesion, mandatory for cell migration, proliferation, and differentiation. Polymeric nanofibres matrices are among the most promising ECM-mimetic biomaterials because their physical structure is similar to that of fibrous proteins in native ECM. They are increasingly being used in TE and have advantages over traditional scaffolds due to increased surface-to-volume ratio, which is supposed to be advantageous for cell-scaffold interaction promoting cellular adhesion, proliferation, migration and function.

Additionally, providing a scaffold made of nanofibres may guide the growth of muscle cells in three dimensions. Attitude and orientation of these fibers are considered to be one of the important features of a functional tissue scaffold containing muscle cells. This leads to the concept of nano-fibrous scaffolds for tissue engineering applications. Further, fiber orientation of the scaffolds greatly influences cell orientation and phenotypic expression [108,109,110]. For instance, Xu *et al*. [107] have evaluated electrospun synthetic biomaterials [poly(L-lactide-co-ε-caprolactone), P(LLA-CL)] using smooth muscle cells. The diameter of the generated fibers was around 500 nm with an aligned topography mimicking the circumferential orientation of cells and fibrils found in the medial layer of a native artery. The results show that the cells adhered and migrated along the axis of aligned scaffolds while expressing a spindle-like phenotype. The cytoskeleton organization inside these cells was also parallel to the orientation of the fibrous assembly. A study by Baker *et al*. showed that smooth muscle cells adapted a more natural organization when grown on electrospun polystyrene scaffolds as compared to collagen fibers *in vivo* [110]. Therefore, engineering scaffolds while controlling the fiber orientation is essential for mimicking structural and functional aspects of the native ECM, controlling cell orientation and tissue growth. Currently, there are a number of methods available for manufacturing tissue scaffolds, which include electrospinning, self-assembly, phase separation, solvent-casting and particulate-leaching, freeze drying, melt molding, template synthesis, drawing, gas foaming, and solid-free forming [27]. Among them, only electrospinning offers the capability to design nanofibrous scaffolds in the form of nonwoven structures that can meet the demands of scaffold-based tissue engineering applications.

### 4.3. Composite Scaffold

Methods to improve cell attachment and proliferation on synthetic materials have already been explored. One approach is to coat a synthetic material with biological substances such as collagen, serum or to use surface modification procedures prior to cell seeding to encourage attachment. *In vitro* cultured SMCs for example have been shown to attach and proliferate extensively on a biodegradable polyesterurethane foam, which was pre-treated with fetal bovine serum [102] as well as on plasma coated, electrospun polystyrene [110].

An alternative approach is to combine different scaffold materials with diverse qualities. The so called composite scaffolds can be fabricated with two or more completely different polymer systems for engineering of hollow organs. Scaffolds designed for hollow organs require a special consideration of their barrier function between the cavity and the surrounding tissues while accommodating sufficient amounts of cells that facilitate tissue development. In the reconstruction of gastrointestinal tissue there is no report of the use of composit scaffolds so far. In bladder reconstruction recent reports using a composite scaffold composed of synthetic PGA and a native acellular matrix (collagen) proved to be optimal for the engineering of bladder tissue combining the advantages of the different materials [111].

Collagen hybrid matrices have been used with mixed results *in vitro*. In one study, PLGA mesh was combined with collagen and processed to become either a sponge or a gel [112]. Cultured porcine urothelial and bladder smooth muscle cells were seeded onto each of the constructs. Smooth muscle cells were able to proliferate and retain expression of differentiation markers when cultured on the on the gel-based construct, but not on the sponge. The opposite was the case for urothelial cells, which stratified on a sponge but not gel, although unequivocal immunohistochemical markers of differentiation were not tested on the urothelium [112]. More promising were the results of Eberli and colleges [111], who used a composite scaffolding system of a native acellular collagen matrix bonded to PGA polymer meshes. The acellular matrix served as a barrier that would prevent the luminal content from permeating into the body cavity while providing an optimal surface for epithelial cell adherence and growth. The synthetic polymer layer with large pores was designed to accommodate sufficient numbers of muscle cells and maintained structural integrity of the scaffold at the same time. The study showed that this composite scaffold remained biocompatible, possessed ideal physical and structural characteristics for hollow organ applications and formed bladder tissue *in vivo* [111]. Composite scaffolds seem to be an ideal approach for the TE of hollow organs, meeting the demands for a biomaterial addressing the unique needs of the different cells used.

## 5. Vascularisation and Innervation

Although many studies demonstrated tissue formation similar to native bladder the functionality of these constructs has never been demonstrated. The two main issues limiting the constructs to be both contractile and capable of physiologic function are proper innervation and vascularisation of the tissue engineered construct.

Rapid neo-vascularisation is essential for graft survival, and complete restoration of the organ structure and functionality. The two main mechanisms forming new blood vessels are angiogenesis (proliferation and migration of endothelial cells from pre-existing vasculature) and vasculogenesis (formation of new vessels by in situ incorporation, differentiation, migration and/or proliferation of endothelial progenerator cells recruited from peripheral blood). Transplanted matrices relay on vascular ingrowth from the surrounding tissue to support previously seeded cells and promote migration of native cells onto the grafted region [14,113].

One of the key factors for engineering of large organs is a vascular network of arteries, veins, and capillaries to deliver sufficient nutrients and oxygen to each cell. One possible method to artificially induce re-vascularisation in engineered tissue might be through application of angiogenic agents such as vascular endothelial growth factor (VEGF) or the implantation of vascular endothelial cells. VEGF is a multifunctional growth factor with mitogenic effects include migration and proliferation of endothelial cells, stabilization of neovessels and tissue remodeling [114]. In addition to its angiogenic function, VEGF also functions as anti-apoptotic factor for smooth muscle [115] and endothelial cells [116].

The positive effect of VEGF on tissue-engineered intestine has been reported by Rocha *et al*. [57]. The engineered tissue was generated by seeding donor neonatal rat intestinal organoid units onto PGA scaffolds along with VEGF containing microspheres. After four weeks *in vivo* the tissue was analyzed showing that sustained delivery of VEGF resulted in up-regulation of microvasculature and epithelial proliferation. The intestinal constructs with VEGF microspheres were significantly larger and epithelial cellular proliferation and capillary density were significantly increased compared to control tissue.

As graft shrinkage seems to be a natural process of a scaffold material for a hollow organ, enhancement of vascular supply to the graft has been conceived as a measure to sustain the viability of regenerated tissue. In bladder augmentation, omental coverage [12], application of exogenous angiogenic growth factors [88,117,118] and endothelial cell seeding [119] were reported to allow ingrowth of capillaries to the graft, but may still be lacking the ability to provide a permanent and sufficiently robust vascular supply to sustain a large graft for the human bladder.

Innervation of the regenerated hollow organ is mandatory for long term functional survival of the graft and to avoid secondary degeneration of organ specific cells. Especially in bladder reconstruction one of the major obstacles for clinical use has been the lack of functional innervation.

Nakase *et al.* were able to induce reinnervation of a tissue engineered small intestine. Nerve fibers extending from the nerve body in the normal tissue to the center of the graft area were observed in regenerated smooth muscle layers, villi and submucosa, but the constitution of myenteric plexus of Auerbach or submucosal plexus of Maissner were not observed.

The influence of different neurotrophic factors in neural development, survival, outgrowth and branching has been investigated by different research groups [120,121,122]. Mitsui *et al*. transplanted immortalized neural stem cells, neuronal and glial restricted precursors, or fibroblasts expressing neurotrophic factors to contused spinal cord, and reported improved bladder function [123]. NGF is the first and best-characterised member of the neurotrophin family. NGF supports survival, outgrowth, and branching of sensory and autonomic neurons, but does not promote motor neuron regeneration [124]. Gene therapy for peripheral nerve regeneration has been used by Sasaki *et al*. They injected nerve growth factor (NGF) to the bladder wall with a replication-defective adenovirus for the treatment of adult diabetic cystopathy and reported a markedly improved bladder function [125]. This viral vector system has been shown to restore decreased NGF expression in the bladder. However, increased levels of NGF in bladder afferent neurons lead to hyperreflexia, which was significantly reduced when NGF levels were neutralized with anti-NGF antibodies [126]. Moreover, high doses of NGF delay nerve regeneration by retarding GAP 43 [127]. Therefore, it is equally important to consider optimal dose and release kinetics for the application of such therapeutic growth factors. Glial cell line-derived neurotrophic factor (GDNF) is a potent survival factor for motor neurons [128]. Variations in GDNF release influenced the rate of functional motor nerve recovery in rat primary peripheral nerve repair [129]. VEGF a potent angiogenetic factor also serves as neurotrophic factor for nerve regeneration [122].

Application of multiple growth factors rather than a single factor may hold great promise to support target organ innervation. The complex neural mechanism regulating bladder function includes various neural subpopulations, which are responsive for different neurotrophic factors. For example, lumbar dorsal root ganglion (DRG) neurons were found to express 65% Ret and 35% TrkA receptors for GDNF and NGF, respectively and 9% of receptors positive for both GDNF and NGF [130]. Madduri *et al*. demonstrated the synergistic effect of GNDF and NGF on axonal elongation and branching form DRG neurons [131].

NGF combined with VEGF enhanced regeneration of bladder acellular matrix grafts in spinal cord injury induced neurogenic rat bladders and protein gene product 9.5 (PGP) positive nerve fibers were observed most abundantly in the groups treated with combined factors rather than single factor treated groups [132]. However, the optimal combination of neurotrophic factors supporting bladder regeneration still remains unclear.

Axonal growth direction is well regulated by topographical features. Longitudinally aligned nanofibres guided the axons unidirectionally compared to random fibres, which showed axonal growth distributed in all directions [133].

## 6. Conclusions

An ideal biomaterial for the engineering of functional tissue for the reconstruction of hollow organs should be biocompatible and support tissue formation as well as provide adequate structural support to the neo-organ during tissue development. Many research groups were able to show engineered constructs structurally similar to native tissue. However, the functionality of these constructs has never been demonstrated. The two main issues hampering the tissue engineered constructs to be contractile and allow normal organ function are proper innervation and vascularisation.

In the near future, tissue engineered scaffolds with controlled topography and multiple neural and angiogenetic factors will provide a potential option to introduce proper biological function to the engineered tissue.
